# Left atrial reverse remodeling in patients with persistent atrial fibrillation and HFpEF following radiofrequency ablation: prospective evaluation with echocardiographic atrial analysis

**DOI:** 10.1016/j.clinsp.2025.100730

**Published:** 2025-08-14

**Authors:** Yan Chen, Mingxia Li, Xiaoxian Wang, Xichen Liang, Minglong Chen, Yingming Zhao, Fen Chen, Yunxian Yu, Cynthia C. Taub, Christopher Lee, Fang Xu, Jing Yao

**Affiliations:** a Department of Cardiology, Jiangsu Province Hospital of Chinese Medicine, Affiliated Hospital of Nanjing University of Chinese Medicine, Nanjing, PR China; b Department of Ultrasound Medicine, Nanjing Drum Tower Hospital, Affiliated Hospital of Medical School, Nanjing University, Nanjing, PR China; c Department of Cardiology, The First Affiliated Hospital of Nanjing Medical University, Nanjing, PR China; d Department of Cardiology, Nanjing Drum Tower Hospital, Affiliated Hospital of Medical School, Nanjing University, Nanjing, PR China; e Department of Epidemiology & Health Statistics, School of Medicine, Zhejiang University, Zhejiang, PR China; f Department of Medicine, Upstate Medical University, Norton College of Medicine, Syracuse, NY, USA; g Division of Cardiology, University of California, San Francisco, San Francisco, CA, USA; h Medical Imaging Center, Nanjing Drum Tower Hospital, Affiliated Hospital of Medical School, Nanjing University, Nanjing, PR China

**Keywords:** Persistent atrial fibrillation, Heart failure with preserved ejection fraction, Radiofrequency catheter ablation, Left atrial strain, Reverse remodeling

## Abstract

**Objective:**

This study aimed to assess the extent of Left Atrial (LA) structural and functional reverse remodeling following Radiofrequency Catheter Ablation (RFCA) for Atrial Fibrillation (AF) in patients with persistent AF and Heart Failure with preserved Ejection Fraction (HFpEF).

**Methods:**

A cohort study was performed following the STROBE Statement. Thirty consecutive HFpEF patients with persistent AF were prospectively enrolled and planned for RFCA. Prior to the procedure, transthoracic echocardiography was performed to assess LA structure, LA strain, and LA contractile function. The same evaluation of LA structure and functional parameters was performed post-ablation at discharge, 1-month, 3-months, 6-months, and 12-months. Baseline data was compared between patients with AF recurrence and non-recurrence post-ablation.

**Results:**

Among the 30 enrolled patients, 4 patients experienced AF recurrence after 3-months, and 7 were lost to follow-up due to the COVID-19 pandemic. Nineteen patients completed the 12-month follow-up without AF recurrence. Structural reverse remodeling was observed in patients without recurrent AF post-ablation, measured by a significant reduction in LA diameters and volumes. LA reservoir Strain (LASres) showed significant improvement at 1-month follow-up, in parallel with the recovery and improvement in LA contractile Strain (LASct). Greater improvement in LA contractile function evaluated via echocardiographic LA imaging parameters was seen in patients who maintained sinus rhythm following ablation.

**Conclusion:**

In patients with persistent AF and HFpEF, RFCA for AF with resultant preservation of sinus rhythm results in the reversal of LA morphological remodeling and notable improvements in LA reservoir and contractile function.

## Highlights

• LA remodeling reversed after successful RFCA in HFpEF patients with persistent AF.
• LA strain parameters effectively assess LA function in HFpEF patients with persistent AF.
• Echocardiographic LA contractile function quantifies reverse remodeling post-RFCA.

## Introduction

Heart Failure with preserved Ejection Fraction (HFpEF) is a common clinical condition, affecting up to 4.9 % of individuals over 60-years of age.^1^ Approximately two-thirds of patients with HFpEF, develop Atrial Fibrillation (AF).^2^ HFpEF and AF share common risk factors and complications, frequently coexisting and mutually exacerbating each other in a deleterious cycle. The presence of persistent AF significantly elevates the likelihood of HFpEF by nearly 40 times.^3^ Compared to patients with HFpEF without AF, patients with both conditions experience a less favorable prognosis and have more pronounced symptomatology.^4^ In recent years, there has been growing interest in transitioning from rate control strategies to rhythm control approaches, particularly in the early stages of AF. Mounting evidence suggests that catheter ablation for AF can improve clinical symptoms and Quality Of Life (QOL) for patients with Heart Failure with reduced Ejection Fraction (HFrEF) by restoring and maintaining sinus rhythm.^5^^,^^6^

The role of ablation in the management of AF in HFpEF patients has attracted increasing attention. Few studies have shown that AF ablation can be effective in improving symptoms and QOL in patients with symptomatic AF and HFpEF.^7^^,^^8^ It is also necessary to objectively evaluate the changes in LA structure and function after ablation in HFpEF patients with persistent AF.

LA morphology and function can be evaluated by cardiac magnetic resonance, cardiac computed tomography, and echocardiography. Echocardiography provides a cost-effective, widely available, and versatile modality to assess the LA with high temporal and spatial resolution.^9^ An increasing amount of data suggests that measures of LA structure and function parameters obtained via echocardiography provide powerful prognostic information for patients with AF and/or HFpEF.^10^^,^^11^ However, a specific evaluation of the value of catheter ablation as a first-line rhythm control therapy in patients with persistent AF has not yet been carried out.^12^ There have been few studies investigating the changes in LA structure and function in patients with persistent AF and HFpEF following Radiofrequency Catheter Ablation (RFCA). Some studies suggest that LA remodeling in patients with persistent AF may be partially irreversible.^13^^,^^14^ The goal of the present study was to assess LA morphological and functional parameters in HFpEF patients with persistent AF before and after RFCA, with a specific focus on analyzing LA functional parameters throughout the follow-up period.

## Methods

### Patient selection and data collection

A total of thirty patients with HFpEF and persistent AF who were unresponsive, intolerant, or unwilling to take antiarrhythmic drugs and had not previously undergone catheter ablation for AF were consecutively recruited at The First Affiliated Hospital of Nanjing Medical University from May to December 2019. Inclusion criteria included patients with a confirmed diagnosis of persistent AF, Left Ventricular Ejection Fraction (LVEF) ≥50 % on echocardiography as measured by the biplane Simpson’s method, and HFA-PEFF score ≥2-points based on the diagnostic algorithm established by the Heart Failure Association of the European Society of Cardiology.^15^ Exclusion criteria included moderate or severe valvular heart disease, myocarditis, cardiomyopathy, coronary artery disease, myocardial infarction, thyroid dysfunction, severe pulmonary hypertension, poorly controlled hypertension, and patients who were unable to follow-up for at least 12-months. Prior to the ablation, a transthoracic echocardiogram and electrocardiogram were performed, with subsequent evaluations at discharge, 1-month, 3-months, 6-months, and 12-months post-ablation, respectively.

### Radiofrequency catheter ablation and follow-up

Prior to RFCA, amiodarone was discontinued for 2-months, and other antiarrhythmic drugs were discontinued for 5 half-lives. Transesophageal echocardiography or LA computed tomography was performed to exclude the presence of LA thrombus prior to ablation. Under the guidance of three-dimensional electrical mapping, LA modeling was performed. In addition to pulmonary vein isolation, linear ablation lesion sets across the mitral isthmus, left atrial roof, and cavo-tricuspid isthmus were performed at the discretion of the operator. All patients received direct oral anticoagulants or warfarin post-ablation for at least 3 months. Follow-up visits were scheduled at discharge, 1st-, 3rd-, 6th-, and 12th-months post-ablation, with a 12-lead Electrocardiogram (ECG) and transthoracic echocardiogram performed at each visit. Electrocardiography was performed immediately when patients presented with any symptoms of suspected recurrent arrhythmia. The recurrence of AF was defined as AF that occurred after the 3-month blanking period, lasting longer than 30 s.

### Echocardiographic image acquisition

Standard transthoracic echocardiography was performed in the left lateral position, using the Vivid E9 ultrasound system equipped with an M5S probe. The ECG was connected synchronously. The 2D grayscale dynamic images were acquired in the parasternal long-axis, apical 4-chamber, 2-chamber, and long-axis views. Left Ventricular End-Diastolic Volume (LVEDV), LV End-Systolic Volume (LVESV), and LVEF were calculated using the modified biplane Simpson’s method in the apical 4- and 2-chamber views. To determine LV Global Longitudinal Strain (GLS), LV endocardial and epicardial borders were automatically traced using automated function imaging in apical 4-, 3- and 2-chamber views, with manual adjustment performed when necessary.^16^ GLS was reported as absolute values. Late transmitral flow (A) and late diastolic peak velocities (a’) of the septal/lateral mitral annulus were recorded using pulse Doppler and tissue Doppler imaging, respectively. All images were stored offline for analysis using commercially available software (Echo-PAC v203 workstation, GE Medical Systems).

### Assessment of LA structural parameters

LA maximal (LAVImax) and minimal (LAVImin) volumes were calculated using the modified biplane Simpson’s rule in apical 4- and 2-chamber views and were indexed to body surface area. Anteroposterior LA Diameters (APLAD) were measured from the parasternal long-axis view, while Transversal (TLAD) and Superior-Inferior (SILAD) LA diameters were assessed at end-systole from the apical 4-chamber view.^17^ Changes in LA volume index and diameter following ablation were determined by calculating the respective percentage changes in LA volume index and diameter as compared to baseline measurements and were assessed at discharge, 1-month, 3-months, 6-months, and 12-months follow-up.

### Assessment of LA phasic functional parameters with 2D speckle tracking

**Fig. 1:**
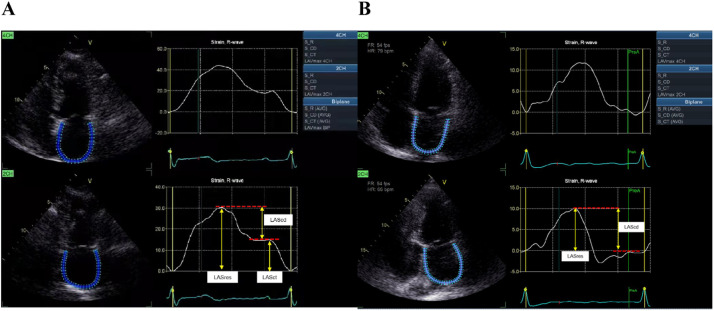
LA strain measurement acquired in the apical 4- and 2-chamber views by 2D-STE in a patient with sinus rhythm (A) and AF (B). LASres, Left Atrial reservoir Strain; LAScd, Left Atrial conduit Strain; LASct, Left Atrial contractile Strain.

LA strain was generated via LA Speckle Tracking Echocardiography (STE). LA endocardial borders were manually traced at end-systole in the apical 4- and 2-chamber views, and strain-time curves were generated automatically (Fig. 1). The R wave, as measured via the synchronous ECG, was utilized to denote end-diastole as the zero-strain reference point.^18^

The LA reservoir Strain (LASres) was calculated as the peak positive value at mitral valve opening. The LA conduit Strain (LAScd) was measured as the absolute value of the difference in strain value between the onset of atrial contraction and mitral valve opening. The LA contractile Strain (LASct), which corresponds with atrial contraction in late diastole that is only observed in sinus rhythm, was measured as the absolute value of the difference in strain value between ventricular end-diastole and the onset of atrial contraction (Fig. 1).^18^

### Assessment of LA contractile function via echocardiographic measures

**Fig. 2:**
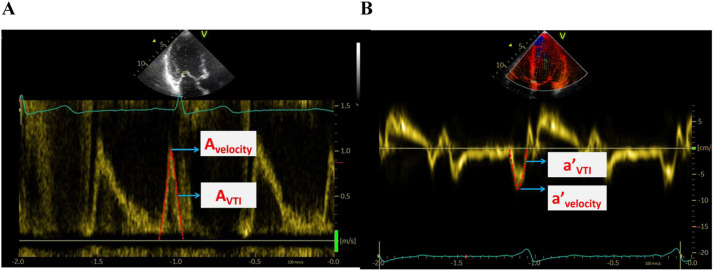
Late transmitral flow A-wave measurement by pulse Doppler (A), and late diastolic a’-wave of the septal/lateral mitral annulus measurement by tissue Doppler imaging (B). A_velocity_, Peak Velocities of transmitral flow A-wave; A_VTI_, Velocity-Time integral of transmitral flow A-wave; a’_velocity_, Peak velocities of septal/lateral mitral annulus a’-wave; a’_VTI_, Velocity-time integral of septal/lateral mitral annulus a’-wave.

LA presystolic Volume (LAVIpre) was measured at the onset of the P wave in ECG using the modified biplane Simpson’s rule in apical 4- and 2-chamber views, indexed to body surface area. LA Active Emptying Fraction (LAAEF) was calculated as (LAVIpre - LAVImin) / LAVIpre*100 %. Peak velocities (A_velocity_) and Velocity-Time Integral (A_VTI_) of transmitral flow A-wave, as well as peak velocities (a’_velocity_) and Velocity-Time Integral (a’_VTI_) of the septal/lateral mitral annulus a’-wave were measured (Fig. 2).

**Fig. 3:**
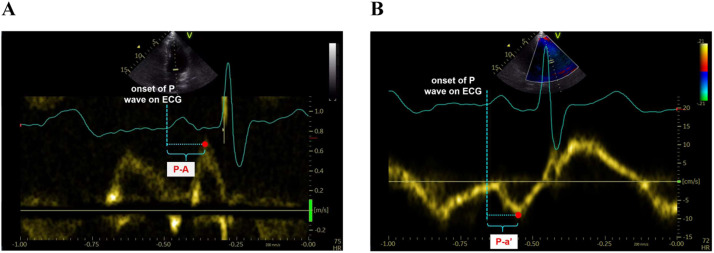
Left atrial electromechanical coupling measurement by pulse Doppler (A), and tissue Doppler imaging (B). P-A, The interval between the onset of the P wave and the peak transmitral flow A-wave; P-a’, The interval between the onset of the P wave and the peak septal/lateral mitral annulus a’-wave.

The interval between the onset of the P wave in ECG and the peak A-wave, as well as the peak a’-wave, was defined as P-A and P-septal/lateral a’, respectively (Fig. 3). All measurements were acquired for 3 cardiac cycles in sinus rhythm and 5 cycles in AF.

### Statistical analysis

Statistical analyses were performed using SPSS 24.0. Categorical data were presented as counts and percentages. Continuous variables were expressed as mean ± standard deviation, and non-normally distributed variables were presented as medians and interquartile range. The Chi-Square or Fisher's exact test was used for comparisons of categorical data. Student's *t*-test was used in comparisons of groups when the data were normally distributed. The Wilcoxon rank sum test was performed if the data were not normally distributed. Repeated measures ANOVA was used in comparison of the alteration in LA diameters and volume, which was calculated as the respective percentage changes; *p* < 0.05 was considered statistically significant in comparison of baseline characteristics, while in pairwise comparisons of LA volume, phasic strain, and contractile function parameters, *p* < 0.01 was considered statistically significant.

## Results

### Baseline clinical and echocardiographic characteristics

A total of 30 patients with persistent AF and HFpEF who underwent AF ablation were enrolled; 4 patients experienced a recurrence of atrial tachyarrhythmias within 3 months following RFCA, and 7 patients were unable to complete follow-up due to COVID-19. Nineteen patients maintained sinus rhythm for a 12-months follow-up post-ablation. The baseline characteristics of each of the groups are summarized in Table 1. No significant differences were observed in age, gender, body mass index, or body surface area between the Recurrence and Non-Recurrence groups. NT-proBNP, blood pressure, and duration of AF were comparable in both groups. Additionally, there were no significant baseline differences in LVEDV, LVESV, LVEF, or LA diameters between the groups. The Non-Recurrence group exhibited significantly smaller LAVImax (46.12 ± 13.57 vs. 53.80 ± 14.06 mL/m^2^, *p* = 0.042) and LAVImin (32.21 ± 11.71 vs. 38.70 ± 10.61 mL/m^2^, *p* = 0.014), higher GLS (12.84 % ± 1.72 % vs. 8.25 % ± 1.65 %, *p* = 0.025) and LASres (13.84 % ± 1.58 % vs. 11.00 % ± 2.08 %, *p* = 0.032) as compared to the Recurrence group. There was no difference in LAScd between the groups.

**Table 1:** Baseline characteristics of the study population.

Characteristics	Recurrence group (*n* = 4)	Non-Recurrence group (*n* = 19)	p-value
Age (years)	63.25 ± 6.40	61.88 ± 8.50	0.76
Gender (male/ %)	2/50	8/42.10	0.67
NT-proBNP (pg/mL)	991.96 ± 740.07	910.54 ± 375.72	0.84
Body mass index (kg/m^2^)	24.54 ± 2.50	23.53 ± 2.89	0.81
Body surface area (m^2^)	1.71 ± 0.12	1.74 ± 0.20	0.73
Systolic Blood Pressure (mmHg)	119.75 ± 14.82	122.32 ± 14.10	0.75
Diastolic Blood pressure (mmHg)	74.25 ± 5.68	81.11 ± 7.79	0.11
AF duration (month)	10.39 ± 10.97	9.50 ± 7.00	0.88
LVEDV (mL)	97.09 ± 21.71	105.72 ± 22.41	0.50
LVESV (mL)	35.99 ± 6.60	40.08 ± 11.13	0.49
LVEF ( %)	63.18 ± 2.27	67.41 ± 10.34	0.43
APLAD (cm)	4.25 ± 0.06	4.02 ± 0.37	0.24
TLAD (cm)	4.68 ± 0.40	4.38 ± 0.47	0.26
SILAD (cm)	5.98 ± 0.10	5.78 ± 0.68	0.24
LAVImax (mL/m^2^)	53.80 ± 14.06	46.12 ± 13.57^a^	0.042
LAVImin (mL/m^2^)	38.70 ± 10.61	32.21 ± 11.71^a^	0.014
LASres ( %)	11.00 ± 2.08	13.84 ± 1.58^a^	0.032
LAScd ( %)	8.75 ± 4.50	11.74 ± 1.66	0.06
GLS ( %)	8.25 ± 1.65	12.84 ± 1.72^a^	0.025

a p < 0.05 vs. Recurrence group.

### LA structural reverse remodeling following ablation in the non-recurrence group

LA dimensions in each direction exhibited no significant differences at discharge compared to their baseline measurements. A notable decrease was observed in the anteroposterior (3.72 ± 0.32 vs. 4.02 ± 0.37 cm, *p* < 0.001) and superior-inferior (5.34 ± 0.59 vs. 5.78 ± 0.68 cm, *p* = 0.006) LA diameters at 1-month follow-up. This decrease in LA size was maintained throughout the subsequent follow-up period. There was no significant change in transverse LA diameter at discharge, 1-month, 3-month, and 6-month follow-up as compared to baseline. At 12-month follow-up, the reduction in TLAD was statistically significant (4.00 ± 0.25 vs. 4.38±0.47 cm, *p* = 0.009) (Table 2).

**Table 2:** Changes in LA structure following RFCA in the non-recurrence group.

	Baseline	Discharge	1-Month	3-Month	6-Month	12-Month
**APLAD (cm)**	4.02 ± 0.37	4.04 ± 0.39	3.72 ± 0.32^a^^,^^b^	3.72 ± 0.41^a^	3.45 ± 0.41^a^	3.54 ± 0.36^a^
**TLAD (cm)**	4.38 ± 0.47	4.36 ± 0.48	4.16 ± 0.44	4.11 ± 0.37	4.01 ± 0.43	4.00 ± 0.25^a^
**SILAD (cm)**	5.78 ± 0.68	5.66 ± 0.52	5.34 ± 0.59^a^	5.15 ± 0.50^a^	5.03 ± 0.38^a^	4.94 ± 0.40^a^
**LAVImax (mL/m^2^)**	46.12 ± 13.57	49.06 ± 13.02	43.40 ± 14.64	38.41 ± 18.25	37.49 ± 8.87	36.56 ± 11.85^a^
**LAVImin (mL/m^2^)**	32.21 ± 11.71	34.29±11.00	25.12 ± 10.84^a^	21.23 ± 8.86^a^	19.90 ± 5.85^a^	18.58 ± 6.88^a^

a p < 0.01 vs. Baseline values.

b *p* < 0.01 vs. Discharge values.

**Fig. 4:**
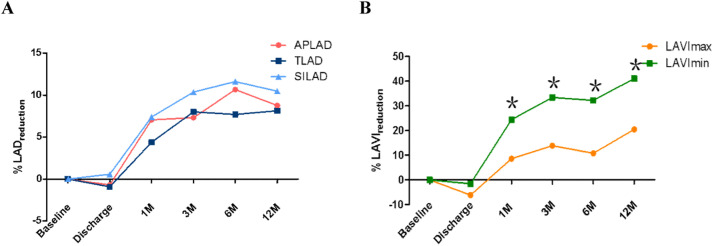
Reduction of anteroposterior, transversal, and superior-inferior LA diameters (A), as well as the maximum and minimum LA volume index (B) from baseline to 12-months follow-up. APLAD, Anteroposterior Left Atrial Diameters; TLAD, Transversal Left Atrial Diameters; SILAD, Superior-Inferior Left Atrial Diameters; LAVImax, Left Atrial maximum Volume Index; LAVImin, Left Atrial minimum Volume Index. **^༊^***p* < 0.01 vs. reduction of LAVImax.

At the 12-month follow-up, the reduction in the superior-inferior LA diameter was greater than that in the anteroposterior and transversal diameters, though these differences were not statistically significant (Fig. 4A).

Compared to baseline measurements, noteworthy reductions in LAVImax were evident until 12-month follow-up (36.56 ± 11.85 vs. 46.12±13.57 mL/m^2^, *p* = 0.006). Significant decreases in LAVImin were observed at 1-month (25.12 ± 10.84 vs. 32.21 ± 11.71 mL/m^2^; *p* < 0.001) following ablation, with further reduction in size during the subsequent follow-up exams (Table 2).

Notably, a more substantial reduction in LAVImin was observed from baseline to the 12-month follow-up when compared to the changes in LAVImax (Fig. 4B).

### Changes in LA strain following ablation in the non-recurrence group

**Fig. 5:**
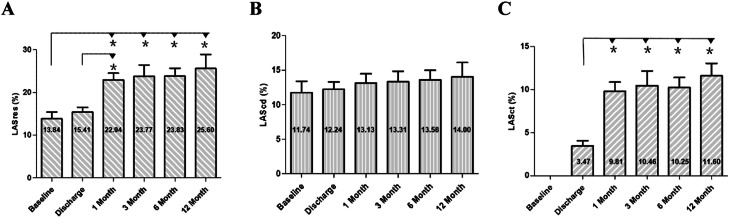
Changes in LA reservoir (A), conduit (B), and contractile (C) strain pre- and post-atrial ablation. LASres, Left Atrial reservoir Strain; LAScd, Left Atrial conduit Strain; LASct, Left Atrial contractile Strain. **^༊^***p* < 0.01 vs. pairwise comparisons among LASres/LASct parameters.

**Fig. 6:**
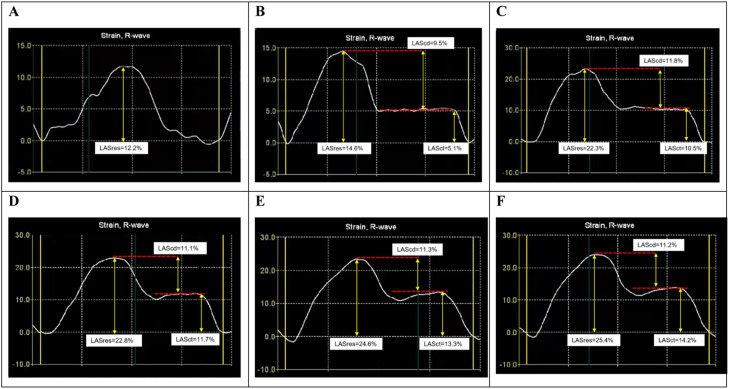
LA strain curve of a representative patient with persistent AF and HFpEF before ablation (A), at discharge (B), 1-month (C), 3-months (D), 6-months (E), and 12-months (F) following ablation. LASres, Left Atrial reservoir Strain; LASct, Left Atrial contractile Strain; LAScd, Left Atrial conduit Strain.

Changes in LA strain parameters were noted in patients without recurrence following RFCA. At discharge, no significant differences were observed in LASres compared to baseline values. However, at 1-month follow-up, LASres exhibited a notable increase (22.94 % ± 1.62 % vs. 13.84 % ± 1.58 %, *p* < 0.001), which persisted throughout the follow-up period. No significant changes were detected in LAScd following ablation. LASct, initially absent in patients with AF, demonstrated recovery post-ablation. A marked increase was observed at 1-month assessment compared to discharge (9.81 % ± 1.07 % vs. 3.47 % ± 0.59 %, *p* < 0.001), with stable values maintained at 3-month and 6-month follow-ups. Although both LASres and LASct showed a slight increase at 12-month follow-up, the difference between the values at 6-months and 12-months did not reach statistical significance (Fig. 5, Fig. 6).

### Changes in LA contractile function evaluated by echocardiographic parameters in the non-recurrence group

LA contractile function parameters were not available at baseline due to AF. All parameters recorded post-ablation exhibited gradual recovery and improvement. At discharge, LAAEF was 16.03 % ± 8.28 %, increasing significantly to 24.71 % ± 7.43 % at 1-month follow-up (*p* = 0.001). Following this initial increase, LAAEF remained stable at this level during the subsequent follow-up periods (Table 3).

**Table 3:** Changes in LA functional parameters measured by Doppler imaging following RFCA in the non-recurrence group.

	**Discharge**	**1-Month**	**3-Month**	**6-Month**	**12-Month**
**LAAEF ( %)**	16.03 ± 8.28	24.71 ± 7.43^a^	28.18 ± 13.08^a^	27.34 ± 13.88^a^	33.69 ± 16.59^a^
**A_velocity_ (cm/s)**	3.71 ± 0.71	5.86 ± 1.01^a^	5.92 ± 1.49^a^	5.30 ± 1.36^a^	5.96 ± 1.25^a^
**A_VTI_ (cm)**	3.82 ± 1.31	5.01 ± 1.54^a^	5.38 ± 1.15^a^	5.57 ± 1.95^a^	5.36 ± 1.26^a^
**P-A (ms)**	123.23 ± 43.69	99.81 ± 34.44	103.54 ± 35.47	103.25 ± 39.42	108.00 ± 64.03
**Septal a’ _velocity_ (cm/s)**	4.54 ± 1.15	6.45 ± 1.35^a^	6.67 ± 1.35^a^	7.10 ± 1.57^a^	7.54 ± 1.80^a^
**Septal a’_VTI_ (cm)**	4.00 ± 1.12	5.22 ± 1.17^a^	5.62 ± 1.19^a^	5.75 ± 1.48^a^	6.00 ± 1.72^a^
**P-septal a’ (ms)**	108.23 ± 42.67	85.13 ± 32.22	82.77 ± 38.89	97.67 ± 28.01	88.70 ± 18.68
**Lateral a’ _velocity_ (cm/s)**	5.37 ± 2.21	6.85 ± 2.17^a^	7.85 ± 2.30^a^	7.79 ± 2.09^a^	7.76 ± 1.66^a^
**Lateral a’ _VTI_ (cm)**	3.35 ± 1.50	5.22 ± 1.56^a^	5.86 ± 1.92^a^	6.17 ± 2.12^a^	6.00 ± 2.14^a^
**P-lateral a’ (ms)**	110.35 ± 20.62	91.69 ± 34.58	98.42 ± 28.72	100.42 ± 25.37	86.90 ± 32.58

a p < 0.01 vs. Discharge values.

Transmitral Doppler late flow (A-wave) demonstrated recovery post-ablation, with peak A wave velocity (A_velocity_) exhibiting a substantial increase at 1-month assessment, compared to discharge (5.86 ± 1.01 vs. 3.71 ± 0.71 cm/s, *p* < 0.001). These values remained stable at the 3-month, 6-month, and 12-month follow-ups. The A-wave Velocity-Time Integral (A_VTI_) recovered at discharge and displayed a significant increase at 1-month follow-up (5.01 ± 1.54 vs. 3.82 ± 1.31 cm, *p* < 0.001), maintaining stability in the subsequent follow-up periods (Table 3). Both the septal a’velocity (6.45 ± 1.35 vs. 4.54 ± 1.15 cm/s, *p* < 0.001) and septal a’_VTI_ (5.22 ± 1.17 vs. 4.00 ± 1.12 cm, *p* < 0.001) showed a marked increase at 1-month assessment, maintaining stable values on subsequent follow-up assessments. Similarly, lateral a’_velocity_ (6.85 ± 2.17 vs. 5.37±2.12 cm/s, *p* = 0.003) and lateral a’_VTI_ (5.22±1.56 vs. 3.35 ± 1.50 cm, *p* < 0.001) exhibited a marked increase at 1-month assessment. A slight decrease, albeit not significant, was observed in P-A, P-Septal a’, and P-Lateral a’ at 1-month follow-up (Table 3).

## Discussion

The primary finding of the current study can be summarized as follows: following successful RFCA in patients with HFpEF and persistent AF, there is reverse left atrial remodeling and improvement in function.

### Persistent atrial fibrillation and HFpEF

HFpEF complicated by AF remains a challenging issue in cardiovascular medicine. AF and HFpEF share similar risk factors and pathophysiological mechanisms, often interacting in a vicious circle.^19^ Elevated LV filling pressures can lead to increased LA pressure and stress, atrial electrophysiological remodeling, and LA fibrosis, ultimately promoting the occurrence of AF.^20^ Conversely, AF can lead to rapid ventricular rates, promoting LV remodeling, cardiomyocyte hypertrophy, fibrosis, and impaired LV diastolic function, subsequently affecting LV systolic function.^21^

Patients with HFpEF and persistent AF generally experience more severe symptoms, worse prognoses, and a higher risk of sudden death compared to HFpEF patients without AF.^4^ Catheter ablation for AF in patients with HFrEF has been demonstrated to be safe and effective in restoring and maintaining sinus rhythm.^22^ AF ablation has also been shown to be effective in improving symptoms and QOL in HFpEF patients with symptomatic persistent AF.^7^^,^^8^ In addition to conventional echocardiographic parameters, a more objective and sensitive method is needed to evaluate LA function in this patient population following atrial ablation.

### Echocardiographic assessment for LA remodeling and reverse remodeling

The left atrium serves as a biomarker for adverse cardiovascular outcomes, particularly in patients with HFpEF and AF. LA enlargement with a subsequent decrease in LA function represents maladaptive structural and functional remodeling, thereby promoting electrical remodeling, which in turn induces further LA structural and functional remodeling. Consequently, a vicious cycle forms in HFpEF patients with AF, entwining LA electrical, structural, and functional remodeling. LA remodeling, defined as a persistent change in LA size and function, is a time-dependent and complex process that is poorly understood.^23^

A significant reduction in LA volume or diameter serves as a surrogate marker for “structural atrial reverse remodeling”. Various noninvasive imaging modalities are utilized for quantifying LA size and structure, with the measurement of LA volume or diameter in different directions utilizing transthoracic echocardiography having the most substantial body of evidence regarding prognostic value.^24^ Studies have associated reverse remodeling of LA maximum and minimum volume post-atrial ablation with fewer AF recurrences.^25^

Assessment of LA functional remodeling is increasingly recognized as a sensitive indicator of cardiovascular disease, necessitating an understanding of its phasic behavior. LA phasic function comprises three distinct phases: reservoir (blood from the pulmonary veins is emptied into the LA during LV systole and the isovolumic states), conduit (blood passively drains from the LA into the LV during early diastole), and contractile (the LA expels approximately 30 % of total stroke volume into the LV during late diastole) phases.

Abnormalities in LA phasic function often manifest earlier than changes in the LV and even before LA enlargement in various disease states.^26^ Particularly, LASres, representing reservoir function, serves as a potent prognostic marker for hospitalization and mortality in heart failure patients.^27^ LASres and LASct predict incidents of AF, even in cases with normal LA size and LV function.^28^ Furthermore, impaired LASres predicts progression from paroxysmal to persistent AF and forecasts AF recurrence after catheter ablation.^29^ LASct corresponds to atrial contraction in late diastole and is only observed in sinus rhythm. Echocardiographic LA imaging parameters, combined with LASct, provide a more comprehensive analysis of LA contractile function. The force of LA contraction during late diastole is assessed using LAAEF, along with parameters derived from the A-wave of the transmitral pulse Doppler and the a’-wave of mitral annular tissue Doppler imaging.

### LA reverse remodeling in HFpEF patients with persistent AF after successful RFCA

Previous studies have illuminated the phenomenon of LA structural reverse remodeling following catheter ablation, indicating a correlation between the restoration of sinus rhythm and atrial structural remodeling.^30^ Similarly, the present study also demonstrated structural reverse remodeling in HFpEF patients with persistent AF after ablation, evidenced by a significant linear decrease in LA dimensions in each echocardiographic view and LA volume index. Notably, the decrease in LAVImin was more immediate and pronounced than that in LAVImax over a 12-month follow-up, underscoring the critical role of LA contractile function restoration post-ablation.

2D-STE enables the quantitative assessment of myocardial deformation by tracking myocardial points in high frame rate 2D gray-scale ultrasound images. The evaluation of LA phasic function using 2D-STE has garnered attention due to its high reproducibility and feasibility.^26^^,^^31^ Parameters such as LASres and LASct provide additional insights into LA function. Increasingly, 2D-STE has been used to assess LA strain to improve diagnostic accuracy and prognosis in multiple clinical settings, particularly in HFpEF.^32^

Prior research has demonstrated impaired LA function in HFpEF, with peak LA strain proving to be a particularly robust measure of LA dysfunction. A lower peak LA strain was associated with a higher prevalence of AF and worse LV systolic and diastolic function.^33^ The results of the present study indicated no significant differences in LASres between baseline and discharge. However, a notable increase was observed at 1-month follow-up, maintaining this level throughout the subsequent follow-up periods.

The partial recovery of atrial stunning after AF ablation has been discovered within about 15 to 30 days, but the time required for recovery of LA function is not clearly defined.^34^ The present study revealed that LASct, LAAEF, the A-wave, and the septal/lateral a’-waves recovered after ablation. At 1-month follow-up, peak velocities, as well as the velocity-time integral of the A-wave and the septal/lateral a’-waves, exhibited a significant increase, indicating improved LA mechanical function. LASct significantly improved and remained relatively stable during the subsequent follow-up periods. Prior studies have demonstrated that LA reservoir strain progressively worsens across HFpEF patients as AF burden worsens.^20^ As found in this study, the improvement of LA reservoir and contractile strain in HFpEF patients with persistent AF was observed by maintaining sinus rhythm after successful ablation.

### Study strengths and clinical application

**Fig. 7:**
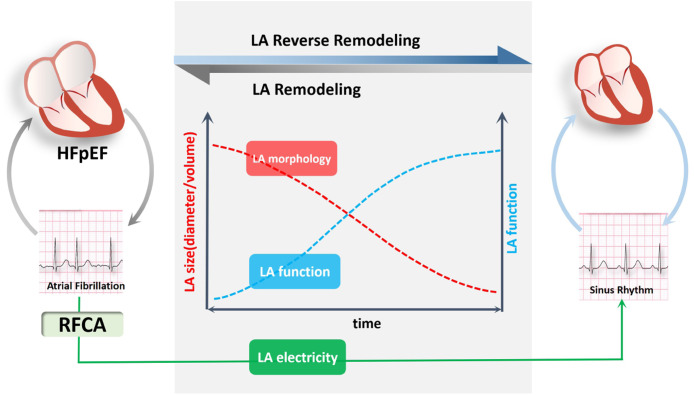
Left Atrial Reverse Remodeling in HFpEF Patients with Persistent AF Following RFCA. HFpEF and AF coexist and mutually exacerbate each other in a vicious cycle. In patients who maintain sinus rhythm after successful RFCA, there is reverse morphological remodeling of the LA and an improvement in LA reservoir and contractile function. Remodeling of LA morphology and function was reversed following LA electrical reverse remodeling by maintaining sinus rhythm after successful RFCA. HFpEF, Heart Failure with preserved Ejection Fraction; RFCA, Radiofrequency Catheter Ablation; AF, Atrial Fibrillation.

In this study, HFpEF patients with persistent AF exhibited reduced LASres and vanishing LASct prior to catheter ablation. In patients who maintained sinus rhythm after successful RFCA, there was a marked improvement in LASres, LASct, LAAEF, as well as peak velocities and the velocity-time integral of the A-wave and the septal/lateral a’-waves. These results suggest that maintaining sinus rhythm contributes to enhanced LA reservoir and contractile function, and is accompanied by structural reverse remodeling of the LA (Fig. 7).

Through a 1-year follow-up, this research carried out a comprehensive and in-depth analysis of the electrical, structural, and functional reverse remodeling of the LA in HFpEF patients with persistent AF after ablation for the first time. Meanwhile, it was discovered that in addition to conventional echocardiographic parameters, the LA phasic function can be regarded as a more sensitive assessment indicator of the LA function in HFpEF patients with persistent AF after ablation.

These results underscore the necessity of focusing on LA functional parameters in HFpEF patients with persistent AF after radiofrequency ablation. Moreover, a systematic assessment of LA reverse remodeling at distinct follow-up intervals is recommended. This study offers valuable evidence to guide clinical decision-making and optimize therapeutic strategies for HFpEF patients with persistent AF.

### Study limitations

This study is subject to several limitations. First, the relatively small number of enrolled patients can be attributed to the strict inclusion criteria and the impact of COVID-19, potentially limiting the generalizability of the results. Second, some differences in baseline characteristics among the two groups might be underestimated due to the relatively small size of the study population. Third, despite conscientious efforts to ensure measurement accuracy, it is crucial to acknowledge potential technical limitations and their impact on the study results. Fourth, there was considerable variability in the selection of linear ablation sites outside bilateral pulmonary vein isolation during ablation. Consequently, it is challenging to investigate the impact of specific ablation targets on LA reverse remodeling in HFpEF patients with persistent AF. Fifth, the follow-up duration was confined to 12 months in the present study. Although the findings offer valuable insights into the short-term effects of radiofrequency ablation on LA and LV function in HFpEF patients with persistent AF, the long-term prognostic significance of LA strain parameters remains to be established. Therefore, further research endeavors, encompassing more extensive and diverse patient cohorts, are imperative to deepen the understanding of LA strain parameters.

## Conclusions

In HFpEF patients with persistent AF, successful radiofrequency ablation and maintenance in sinus rhythm have demonstrated the capability to reverse morphological remodeling of the LA and mitigate damage to LA function. LA strain parameters have emerged as effective tools for assessing LA function in this patient population. Furthermore, the integration of echocardiographic LA contractile function assessment offers objective and sensitive quantitative indicators for evaluating the reverse remodeling seen after ablation.

## Glossary

A_velocity_, Transmitral A-wave peak velocity; A’_velocity_, Septal/lateral mitral annulus a’-wave peak velocity. A_VTI_, Transmitral A-wave velocity-time integral; A’_VTI_, eptal/lateral mitral annulus a’-wave velocity-time integral; AF, Atrial fibrillation; APLAD, Anteroposterior Left Atrial Diameter; ECG, Electrocardiogram; GLS, Global Longitudinal Strain; HFpEF, Heart failure with preserved Ejection Fraction; HFrEF, Heart Failure with reduced Ejection Fraction; LA, Left Atrium; LAAEF, Left Atrial Active Emptying Fraction; LAScd, Left Atrial conduit Strain; LASct, Left Atrial contractile Strain; LASres, Left Atrial reservoir Strain; LAVImax, Left Atrial maximal Volume Index; LAVImin, Left Atrial minimal Volume Index; LAVIpre, Left Atrial presystolic Volume Index; LV, Left Ventricle; LVEDV, Left Ventricular End-Diastolic Volume; LVEF, Left Ventricular Ejection Fraction; LVESV, Left Ventricular End-Systolic Volume; QOL, Quality Of Life; RFCA, Radiofrequency Catheter Ablation; SILAD, Superior-Inferior Left Atrial Diameter; STE, Speckle Tracking Echocardiography; TLAD, Transverse Left Atrial Diameter.

## Ethics approval

This study followed the ethical guidelines stated in the Declaration of Helsinki. This study was approved by the Ethics Committee of The First Affiliated Hospital of Nanjing Medical University (nº 2015-SR-085), and all patients provided informed consent.

## Declaration of generative AI and AI-assisted technologies in the writing process

The authors declare that there is no use of generative AI and AI-assisted technologies in the writing process.

## Research data for this article

Due to the sensitive nature of the questions asked in this study, survey respondents were assured that raw data would remain confidential and would not be shared.

## Authors’ contributions

All authors contributed to the study's conception and design. Data curation and formal analysis were performed by Yan Chen, Mingxia Li, Xiaoxian Wang, Xichen Liang, Minglong Chen, Yingming Zhao, and Fen Chen. Yunxian Yu, Cynthia C. Taub, and Christopher Lee contributed to the supervision and validation of the research. Yan Chen and Mingxia Li wrote the original draft. Fang Xu and Jing Yao contributed to the review and editing of the manuscript. All authors read and approved the final manuscript.

## Funding

This work was supported by grants from the National Natural Science Foundation of China (nº 81,771,844, nº 82,371,981) and Clinical Trials from the Affiliated Drum Tower Hospital, Medical School of Nanjing University (nº 2023-LCYJ-MS-21) to Dr. Jing Yao.

## Declaration of competing interest

The authors declare no conflicts of interest.
